# A prediction model for outcome in patients with HBV-ACLF based on predisposition, injury, response and organ failure

**DOI:** 10.1038/s41598-020-77235-3

**Published:** 2020-11-19

**Authors:** Fangfang Liu, Zhengsheng Zou, Lijun Shen, Weiwei Wu, Jiajun Luo, Seth Lankford, Yongli Yang, Huang Huang, Shaoli You, Bing Zhu, Jin Li, Jinsong Mu, Yawei Zhang, Shaojie Xin

**Affiliations:** 1 Liver Disease Department, Fifth Medical Center of Chinese PLA General Hospital, Beijing, China; 2 Medical School of Chinese PLA, Beijing, China; 3 Department of Epidemiology, School of Public Health, Shanxi Medical University, Taiyuan, Shanxi China; 4 Department of Environmental Health Sciences, Yale School of Public Health, Yale University, New Haven, CT USA; 5 Duke University, Durham, NC USA; 6 Department of Epidemiology and Health Statistics, College of Public Health, Zhengzhou University, Zhengzhou, Henan China; 7 Critical Care Department, Fifth Medical Center of Chinese PLA General Hospital, Beijing, China; 8 Department of Surgery, Yale School of Medicine, Yale University, New Haven, CT USA

**Keywords:** Gastroenterology, Hepatology, Liver diseases

## Abstract

We aimed to develop a prediction model based on the PIRO concept (Predisposition, Injury, Response and Organ failure) for patients with Hepatitis B Virus (HBV) related acute-on-chronic liver failure (ACLF). 774 patients with HBV related ACLF defined in the CANONIC study were analyzed according to PIRO components. Variables associated with mortality were selected into the prediction model. Based on the regression coefficients, a score for each PIRO component was developed, and a classification and regression tree was used to stratify patients into different nodes. The prediction model was then validated using an independent cohort (n = 155). Factors significantly associated with 90-day mortality were: P: age, gender and ACLF type; I: drug, infection, surgery, and variceal bleeding; R: systemic inflammatory response syndrome (SIRS), spontaneous bacteria peritonitis (SBP), and pneumonia; and O: the CLIF consortium organ failure score (CLIF-C OFs). The areas under the receiver operating characteristics curve (95% confidence interval) for the combined PIRO model for 90-day mortality were 0.77 (0.73–0.80). Based on the scores for each of the PIRO components and the cut-offs estimated from the classification and regression tree, patients were stratified into different nodes with different estimated death probability. Based on the PIRO concept, a new prediction model was developed for patients with HBV related ACLF, allowing stratification into different clusters using the different scores obtained in each PIRO component. The proposed model will likely help to stratify patients at different risk, defining individual management plans, assessing criteria for specific therapies, and predicting outcomes.

## Introduction

Acute-on-chronic liver failure (ACLF) is characterized by an acute deterioration in liver function with pre-existing chronic liver disease. It could happen due to a hepatic or extrahepatic injury or an unknown reason. ACLF may result in multisystem organ failure and is associated with significant morbidity and mortality. High mortality (> 50%) has persisted for this condition over the past 20 years^1^.

There are multiple definitions for ACLF proposed by various organizations and studies, including the Asia–Pacific Association for the Study of the Liver (APASL)^2^, the European Association for the Study of the Liver (EASL) and the American Association for the Study of Liver Diseases (AASLD)^3^, the European Association for the Study of the Liver-chronic liver failure (EASL-CLIF) Consortium Acute oN chrONIC liver failure study (CANONIC study)^4^, and the North American Consortium for the Study of End-Stage Liver Disease (NACSELD study)^5^. The most recent definition of ACLF was defined in the CANONIC study^4^. They also proposed a theory to divide ACLF into three categories depending on the underlying liver diseases: type A (without cirrhosis), type B (with compensated cirrhosis), and type C (with decompensated cirrhosis)^1^.

Scoring systems have been used for prediction of prognosis of patients with ACLF, such as the Child-Turcotte-Pugh score^6^, the Model for End-stage Liver Disease score (MELDs)^7^, the Sequential Organ Failure Assessment score (SOFAs)^8^, the Chronic Liver Failure-SOFA score (CLIF-SOFAs)^4^, the CLIF Consortium Organ Failure score (CLIF-C OFs)^9^, and the Chronic Liver Failure Consortium ACLF score (CLIF-C ACLFs)^9^. Jalan et al., developers of CLIF-C ACLFs, found that CLIF-C ACLFs outperformed other scoring systems in prognostic accuracy^9^. However, CLIF-C ACLFs was developed based on the CANONIC study, a multiple site study conducted in 29 European hospitals where patients had an extremely low rate of hepatitis B virus (HBV) infection^4^. It raises a concern whether these scoring systems will have prognostic accuracy in ACLF patients with HBV infection, which accounts for a majority of the ACLF patients in eastern countries^2^.

PIRO concept (Predisposition, Injury, Response, Organ), being used as a scoring system in patients with sepsis^10,11^, could comprehensively examine the situation of ACLF. Among ACLF patients, predisposition (P) could be defined as severity of chronic liver disease evaluated through demographics, and ACLF type and MELDs; injury (I) could be explained by the precipitating event including hepatic (i.e., virus, drugs use, alcohol, etc.) and/or extrahepatic events (i.e., infection and variceal bleeding); response (R) was the body reaction to the injuries (i.e., inflammation); and organ (O) was defined as organ failure^1^.

Here, we conducted a study (1) to develop a new scoring system based on the PIRO concept among ACLF patients with HBV infection, (2) to stratify the patients with different mortality into different clusters using classification tree according to the above scoring system, and (3) to set up a calculator for patients to calculate their own estimated death probability using the above scoring system.

## Methods

### Study design

Patients were screened and enrolled from January 2012 to October 2017 in the Fifth Medical Center of Chinese PLA Hospital (Beijing, China) after the appropriate approvals were obtained. A total of 1,887 consecutive ACLF patients aged 18 to 64 were admitted to the hospital during the study period. After exclusion of patients (N = 673) who had one or more of the following conditions: HCV infection (N = 30), alcohol liver disease (N = 133), autoimmune liver disease (N = 21), drug-induced liver injury (N = 27), Wilson disease (N = 1), cryptogenic liver disease (N = 113), hepatocellular carcinoma (N = 185), other site carcinoma (N = 11), refused to participate the study (N = 158), and liver transplant (N = 39), a total of 1214 patients were included according to the APASL criteria for ACLF^2^. 774 patients were finally enrolled in this study according to the diagnostic criteria for ACLF in the CANONIC study^4^. Causes of exclusion are summarized in Figure S1. We randomly selected 619 patients as the training cohort and 155 patients as the validation cohort. All study procedures were approved by the Institutional Review Board at Fifth Medical Center of Chinese PLA Hospital. Written informed consent was obtained from each patient or his/her legal surrogate before enrollment.

### Definitions

ACLF was diagnosed according to the CLIF Consortium Organ Function score (CLIF-C OFs)^9^. We also categorized ACLF patients into three groups^1^: type A (without cirrhosis), type B (with compensated cirrhosis), and type C (with decompensated cirrhosis).

Chronic HBV infection^12^ was defined as hepatitis B surface antigen (HBsAg) positive and antibody to hepatitis B core antigen (anti-HBc) positive. Chronicity was defined by the presence of HBsAg positive for > 6 months. Cirrhosis was diagnosed by liver biopsy, endoscopic signs of portal hypertension, radiological evidence of liver nodularity, or clinical evidence of previous hepatic decompensation (including ascites, hepatic encephalopathy (HE), and acute variceal bleeding) in patients with chronic liver diseases^5^.

Organ failure was defined according to the CLIF-C OF scoring system^9^ (Table S1). Ascites was diagnosed by clinical examination and confirmed by ultrasonography^13^. HE was defined and graded by West-Haven criteria^14^. Acute variceal bleeding was diagnosed by Baveno V endoscopic criteria^15^. We defined infections according to standard criteria^16^ as follows: (1) positive blood cultures in the absence of any recognized source of infection (spontaneous bacteremia); (2) spontaneous bacterial peritonitis (SBP) was diagnosed in presence of ascitic fluid absolute neutrophil count > 250 cells/mm^3^^17^; (3) radiographic evidence of pulmonary infiltration associated with purulent sputum (pneumonia); (4) urinary WBC count > 15 cells per high-power field and positive urine culture (urinary tract infection). The systemic inflammation response syndrome (SIRS) was assessed according to the recommendations of the American College of Chest Physicians/Society of Critical Care Medicine Consensus Conference^18^. Patients were considered to have SIRS if they fulfilled at least 2 of the following criteria: (1) a core temperature of > 38 °C (100.4 °F) or < 36 °C (96.8 °F); (2) a heart rate of ≥ 90  beats/min; (3) a respiratory rate of ≥ 20  breaths/min; or (4) a white blood cell (WBC) count of ≥ 12,000/mm^3^ or ≤  4000/mm^3^, or a differential count showing ≥ 10% immature polymorph nuclear neutrophil cells (PMNC). Patients with SIRS and infection were considered to have sepsis.

### Data collection

We collected the following clinical and demographic information: age, gender, underlying liver disease, clinical presentation, laboratory parameters, and outcomes. Baseline clinical characteristics were obtained within 48 h of admission to the hospital or, when indicated, at the time of diagnosis of ACLF. For patients discharged from the hospital, prognostic information was obtained from medical records and telephone contact.

### Prognostic scores of ACLF

MELD score was calculated as follows: MELDs = 9.6 × ln [creatinine (mg/dl)] + 3.8 × ln [bilirubin (mg/dl)] + 11.2 × ln (INR) + 6.43 × (etiology: 0 if cholestatic or alcoholic, 1 otherwise)^7^. CLIF-C OFs was summarized based on criteria listed in Table S1^9^. CLIF-C ACLFs was calculated as follows: CLIF-C ACLFs = 10 × [0.33 × CLIF-C OFs + 0.04 × Age + 0.63 × ln (WBC count)-2]^9^.

We used the PIRO concept to build up the scoring system. Variables were grouped according to each PIRO component. “P” was evaluated by age, gender, ACLF type. “I” was characterized by hepatic injury (superimposed on other viruses, alcohol, and drug) and/or extrahepatic injury (surgery, infection, and variceal bleeding). “R” was defined by SIRS, SBP, bacteremia, urinary tract infection, and pneumonia. “O” was assessed by CLIF-C OFs.

### Statistical analysis

Continuous variables were described as mean ± standard deviation (SD). Comparisons between two groups were conducted using Student’s t-test or ANOVA for continuous variables when homogeneity of variance is valid, or else using correction t-test or ANOVA. Chi-square test was used for categorical variables when homogeneity of variance is valid, or else using continuity adjusted Chi-Square.

We randomly selected the training cohort (80%) and validation (20%) cohort from the total population. Firstly, we set up the scoring system and prognostic model using the training cohort. Secondly, we used the validation cohort to validate the prognostic accuracy of the scoring system and prognostic model obtained from the training cohort. Finally, we set up the scoring system and prognostic model using the total population in order to increase the prognostic power.

Variables that were associated with mortality in the univariate analysis (p value < 0.5) were screened for the multivariate models. Four separate multivariate logistic regression models, one for each PIRO component (P, I, R and O) with 90-day mortality, were built using stepwise selection on the variables screened in the univariate analysis. Once the models were fitted, the set of four scores from regression coefficients were generated for each patient corresponding to 90-day mortality. Then the set of four scores were included in the final combined PIRO model. We used this logistic regression model to generate the calculator for each patient to predict his/her 90-day death probability. Odds ratio (OR) with 95% confidence interval (CI) were calculated to estimate the associations between various predictive variables and mortality. The area under receiver operating characteristics curve (AU-ROC) was employed to assess the accuracy of the models. Calibration was tested using the Hosmer–Lemeshow goodness-of-fit test.

To simplify the computation of the scoring system, the regression coefficients were uniformly rescaled to make the maximum total score 50. The AU-ROCs of the models generated by the simplified scoring systems were identical to those derived from the original regression coefficients.

A classification and regression tree was used to define cut-offs for each PIRO component using the new scores. Each node split decision in the tree was chosen from the possible cut-offs for all components according to Gini’s coefficient impurity measure. Each node contained at least 5 alive or dead patients. One sample Z test was used for the comparison of mortality rates between validation cohort and estimated mortality rates obtained from training cohort.

All analysis used a two-sided P-value of 0.05 as statistical significance. Statistical analyses were performed using SAS 9.4 (SAS Institute Inc. Cary. NC. USA).

### Ethical approval

All procedures performed in studies involving human participants were in accordance with the ethical standards of Fifth Medical Center of Chinese PLA General Hospital research committee and with the 1964 Helsinki declaration and its later amendments or comparable ethical standards.

### Informed consent

Informed consent was obtained from all individual participants included in the study.

## Results

### Characteristics of patients with HBV related ACLF in training and validation cohorts

Among patients in training cohort (Table 1), the mean (SD) age was 46.98 (9.65) years and most patients (84.98%) were male. The means (SD) of MELDs, CLIF-C OFs and CLIF-C ACLFs were 26.72 (6.25), 9.25 (1.43) and 41.63 (7.27), respectively. The mortality rate of 90-day was 73.51%. The validation cohort patients showed similar characteristics as the training cohort (P > 0.05).

**Table 1:** Comparison of characteristics between training cohort and validation cohort.

Variables	Training cohort (n = 619)		Validation cohort (n = 195)		P value
	Mean	SD	Mean	SD	
Age	46.98	9.65	45.74	10.00	0.16
MELDs	26.72	6.25	26.45	6.40	0.64
CLIF-C OFs	9.25	1.43	9.17	1.37	0.53
CLIF-C ACLFs	41.63	7.27	40.49	7.01	0.08

	Number	%	Number	%	
Male gender	526	84.98	133	85.81	0.8
90-day mortality	455	73.51	114	73.55	0.99

*ACLF* acute-on-chronic liver failure, *MELDs* model for end-stage liver disease score, *CLIF-OFs* CLIF consortium organ failure score, *CLIF-C ACLFs* chronic liver failure consortium ACLF.

We set up a clinical scoring system based on PIRO concept and a prediction model for 90-day death probability using the training cohort. The clinical scoring system obtained from the training cohort was validated in an independent validation cohort, which provided similar patterns of death probability distribution in the tree nodes. To increase the prognostic power, we used the total population to build the clinical scoring system based on PIRO concept and a prediction model for 90-day death probability.

### Scoring system based on PIRO concept using total population

Distributions of characteristics in each PIRO component by 90-day mortality and ACLF type were shown in Tables 2 and 3, respectively. In the total population, 601 (77.65%) patients were treated with nucleic acid analogs. The reasons some patients did not use nucleic acid analogs were as follows: 1. Patients who met the indications for anti-HBV therapy failed to see a doctor in time for standardized treatment, especially in the areas with poor medical standards. 2. Patients did not meet the indications for anti-HBV therapy before their acute exacerbation of hepatitis B due to injuries, such as infection, surgery, abuse of alcohol or drugs. Age, gender, ACLF type, drug, infection, surgery, variceal bleeding, SIRS, SBP, pneumonia, CLIF-C OFs were associated with 90-day mortality.

**Table 2:** Characteristics in PIRO components with 90-day mortality.

	Total (n = 774)		Survivor (n = 205)		Non-survivors (n = 569)		P
	Number	%	Number	%	Number	%	
**Predispositon ("P")**							
Age (years)							
≤ 50	490	63.31	144	70.24	346	60.81	
> 50	284	36.69	61	29.76	223	39.19	0.0179
Gender							
Male	659	85.14	172	83.90	487	85.59	
Female	115	14.86	33	16.10	82	14.41	0.56
ACLF type							
A	212	27.39	94	45.85	118	20.74	
B	458	59.17	99	48.29	359	63.09	
C	104	13.44	12	5.85	92	16.17	< 0.0001
**Injury ("I")**							
Hepatic							
Virus	99	12.79	29	14.15	70	12.30	0.5
Alcohol	168	21.71	47	22.93	121	21.27	0.62
Drug	56	7.24	9	4.39	47	8.26	0.067
Extrahepatic		0.00					
Infection	100	12.92	19	9.27	81	14.24	0.069
Surgery	14	1.81	1	0.49	13	2.28	0.18*
Variceal bleeding	20	2.58	2	0.98	18	3.16	0.091
**Response ("R")**							
SIRS	154	19.90	28	13.66	126	22.14	0.0091
Infection	434	56.07	90	43.90	344	60.46	< 0.0001
SBP	291	37.60	67	32.68	224	39.37	0.09
Bacteremia	78	10.08	14	6.83	64	11.25	0.072
Urinary tract infection	49	6.33	11	5.37	38	6.68	0.51
Pneumonia	171	22.09	15	7.32	156	27.42	< 0.0001
**Organ failure ("O")**							
CLIF-C OF score							
6–8	235	30.36	102	49.76	133	23.37	
9	175	22.61	44	21.46	131	23.02	
10–18	364	47.03	59	28.78	305	53.60	< 0.0001
**Anti-HBV therapy**							
Nucleic acid analogue			162	79.02	439	77.15	0.6256*
**Other scoring systems**			Mean	SD	Mean	SD	
MELDs			24.04	4.38	26.59	6.22	< 0.0001
CLIF-OFs			8.31	1.08	9.12	1.38	< 0.0001
CLIF-C ACLFs			37.01	6.10	41.32	7.10	< 0.0001

*ACLF* acute-on-chronic liver failure, *CLIF-OFs, CLIF* consortium organ failure score, *CLIF-C ACLFs* chronic liver failure consortium ACLF.

*Continuity adjusted chi-square.

**Table 3:** Characteristics in PIRO components with different ACLF type.

	ACLF type A (n = 212)		ACLF type B(n = 458)		ACLF type C (n = 104)		P
	Number	%	Number	%	Number	%	
**Predispositon ("P")**							
Age (years)							
≤ 50	158	74.53	286	62.45	46	44.23	
> 50	54	25.47	172	37.55	58	55.77	< 0.0001
Gender							
Male	174	82.08	404	88.21	81	77.88	
Female	38	17.92	54	11.79	23	22.12	0.0095
**Injury ("I")**							
Hepatic							
Virus	25	11.79	49	10.70	25	24.04	0.001
Alcohol	40	18.87	104	22.71	24	23.08	0.4991
Drug	21	9.91	33	7.21	2	1.92	0.0364
Extrahepatic							
Infection	15	7.08	62	13.54	23	22.12	0.0007
Surgery	5	2.36	6	1.31	3	2.88	0.4317
Variceal bleeding	1	0.47	11	2.40	8	7.69	0.0007
**Response ("R")**							
SIRS	32	15.09	98	21.40	24	23.08	0.1122
Infection							
SBP	50	23.58	190	41.48	51	49.04	< 0.0001
Bacteremia	20	9.43	47	10.26	11	10.58	0.9311
Urinary tract infection	13	6.13	27	5.90	9	8.65	0.5749
Pneumonia	29	13.68	109	23.80	33	31.73	0.0005
**Organ failure ("O")**							
CLIF-C OF score							
6–8	76	35.85	38	8.30	98	94.23	
9	125	58.96	109	23.80	224	215.38	
10–18	34	16.04	28	6.11	42	40.38	0.0825
**Anti-HBV therapy**							
Nucleic acid analogue	172	81.13	347	75.76	82	78.85	0.2858
**Other scoring systems**	Mean	SD	Mean	SD	Mean	SD	
MELDs	25.51	6.07	27.10	6.10	27.11	7.17	0.0072
CLIF-OFs	9.12	1.46	9.30	1.38	9.17	1.43	0.2827
CLIF-C ACLFs	40.11	6.96	41.73	7.26	42.60	7.32	0.005

*ACLF* acute-on-chronic liver failure, *CLIF-OFs,CLIF* consortium organ failure score, *CLIF-C ACLFs* chronic liver failure consortium ACLF.

Multivariate analysis showed that these variables were independently associated with mortality (Table 4). Variables retained in the final models included age, gender and ACLF type for P, drug, infection, surgery, and variceal bleeding for I, SIRS, SBP, and pneumonia for R, and CLIF-C OFs for O. ACLF type showed a higher performance in predicting 90-mortality (P < 0.0001).

**Table 4:** Selected variables in multivariate analysis.

	90-day mortality			
	OR	95%CI		P value
		Lower	Upper	
**Predispositon ("P")**^**a**^				
Age				
≤ 50	Ref			
> 50	1.30	0.90	1.88	0.17
Gender				
Male	Ref			
Female	1.21	0.75	1.95	0.44
ACLF type				
A	Ref			
B	2.78	1.95	3.96	< .0001
C	5.72	2.93	11.15	< .0001
**Injury ("I")**^**b**^				
Hepatic				
Drug	1.95	0.93	4.06	0.076
Extrahepatic				
Infection	1.57	0.92	2.67	0.096
Surgery	3.86	0.50	30.09	0.2
Variceal bleeding	3.03	0.69	13.28	0.14
**Response ("R")**^**c**^				
SIRS	1.48	0.93	2.34	0.096
Infection				
SBP	1.24	0.88	1.75	0.22
Pneumonia	4.42	2.52	7.76	< .0001
**Organ failure ("O")**				
CLIF-C Ofs				
< 9	Ref			
9	2.28	1.49	3.50	0.0002
> 9	3.97	2.71	5.80	< .0001

*ACLF* acute-on-chronic liver failure, *MELDs* model for end-stage liver disease score, *CLIF-OFs* CLIF consortium organ failure score, systemic inflammatory response syndrome, *SBP* spontaneous bacteria peritonitis, *OR* odds ratio, *Ref.* reference group for OR.

^a^Adjusted for gender.

^b^Adjusted for superimposing other virus and alcohol drinking.

^c^Adjusted for urinary tract infection.

### Performance of PIRO model for predicting 90-day mortality

**Figure 1:**
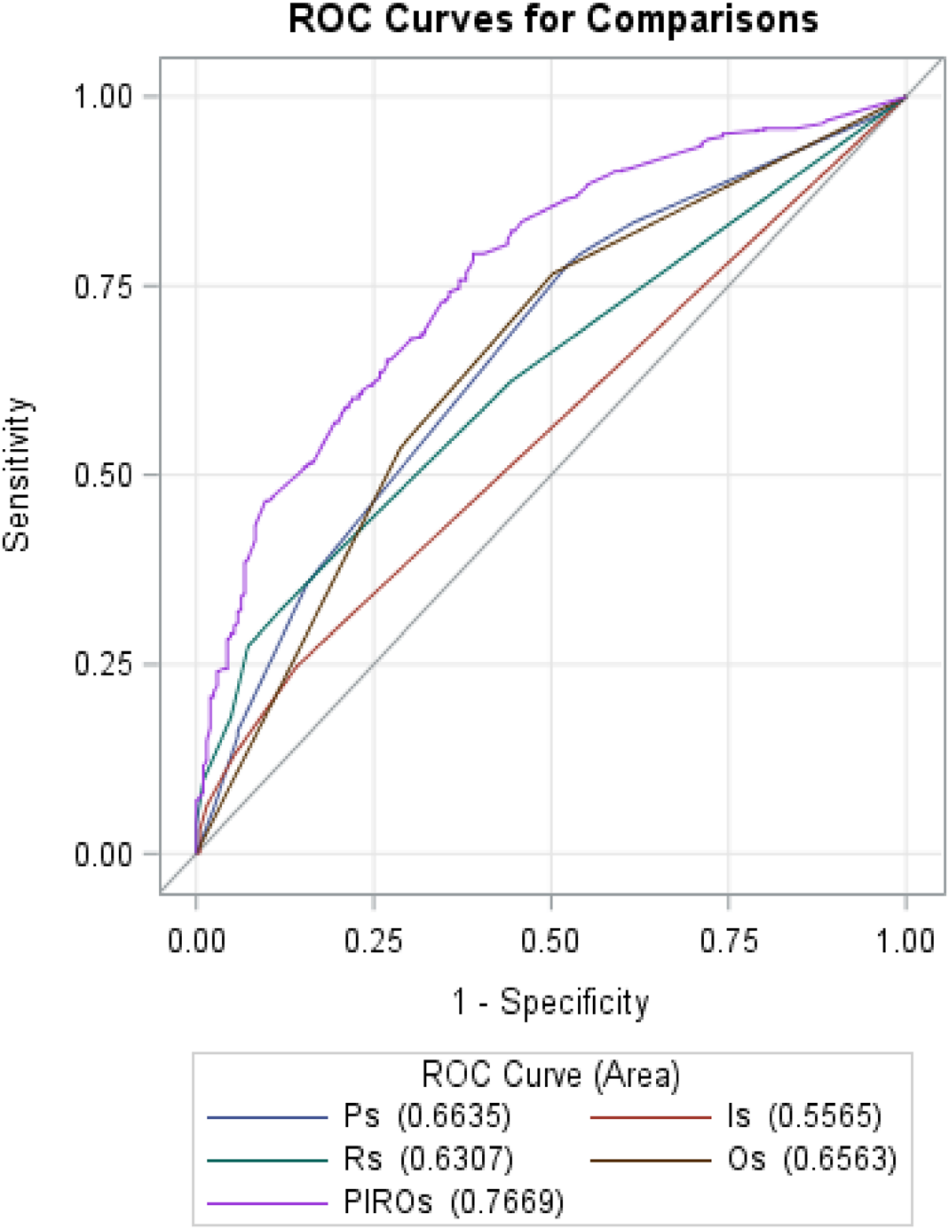
Accuracy of the PIRO score as compared to each component of PIRO in predicting 90-day mortality of patients with HBV-ACLF.

**Figure 2:**
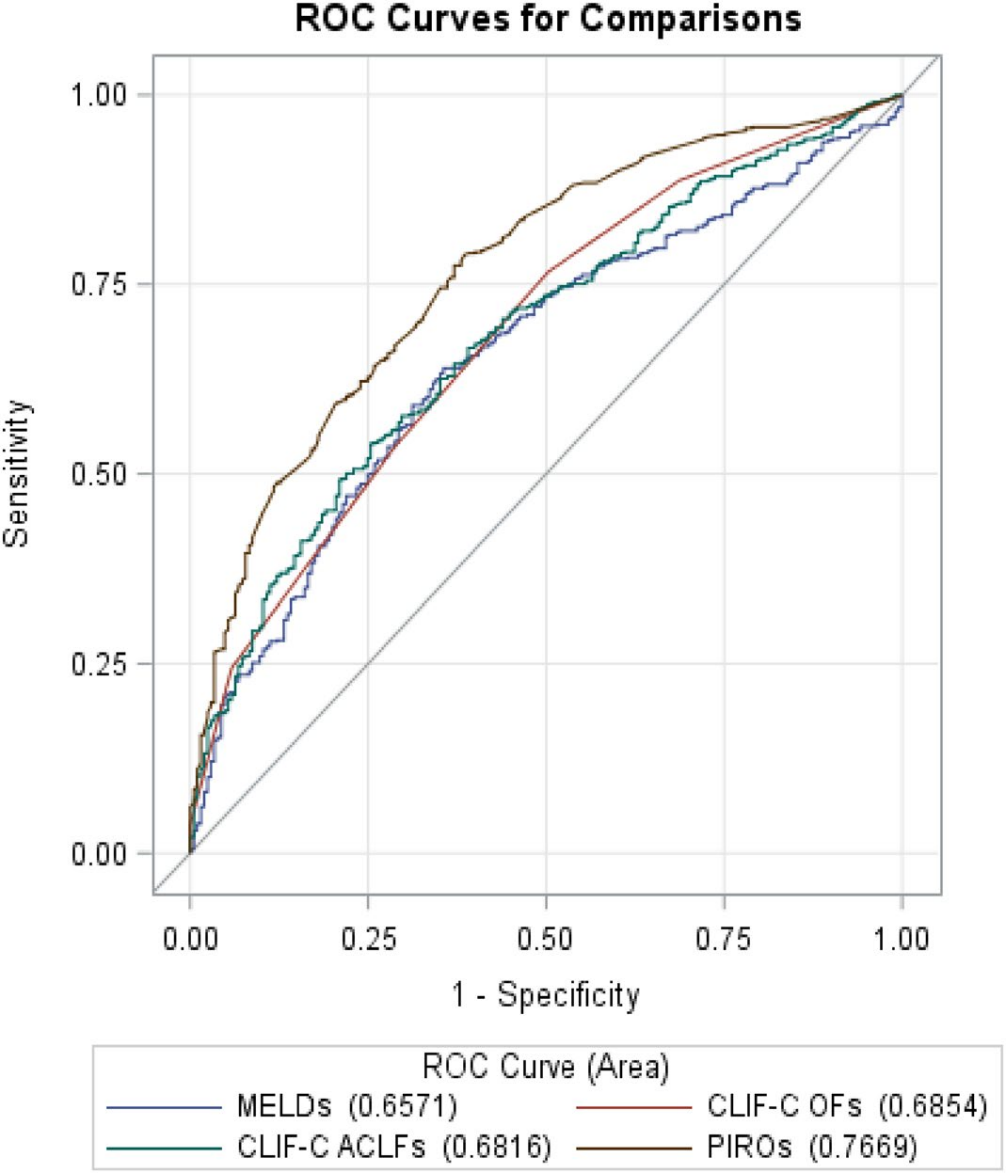
Accuracy of the combined PIRO score as compared to other scoring systems in predicting 90-day mortality of ACLF patients with HBV infection.

The combined PIRO model for 90-day mortality had an AU-ROC (95% CI) of 0.77 (0.73–0.80) (Table 5 and Fig. 1). We also compared the predictive ability of combined PIRO model to current traditional MELDs, CLIF-C OFs, and CLIF-C-ACLFs. The AU-ROC (95% CI) of combined PIRO was higher than MELDs of 0.66 (0.62–0.70), CLIF-C OFs of 0.69 (0.64–0.73), and CLIF-C ACLFs of 0.68 (0.64–0.72) for 90-day mortality (P < 0.0001) (Fig. 2). The Hosmer and Lemeshow test did not show evidence for lack of fit in MELDs, CLIF-C OFs, CLIF-C ACLFs, all four PIRO components, and the combined PIRO models in the training or validation cohorts (p > 0.05).

**Table 5:** Area under the receiver operating characteristics curve (AU-ROC) (95% confidence interval, CI) for predicting 90-day mortality.

90-day mortality	AUROC	P value
**a. AUROC (95% CI) of 90-day mortality by each PIRO component and the combined PIRO model**		
Predisposition ('P')	0.66 (0.62–0.70)	< 0.0001
Injury ('I')	0.56 (0.53–0.59)	< 0.0001
Response('R')	0.63 (0.59–0.67)	< 0.0001
Organ failure ('O')	0.66 (0.61–0.70)	< 0.0001
PIRO	0.77 (0.73–0.80)	Ref
**b. AUROC (95% CI) of mortality by combined PIRO model and other scoring systems**		
MELDs	0.66 (0.62–0.70)	< 0.0001
CLIF-OF	0.69 (0.64–0.73)	< 0.0001
CLIF-C ACLFs	0.68 (0.64–0.72)	< 0.0001
PIRO	0.77 (0.73–0.80)	Ref

*MELDs* model for end-stage liver disease score, *CLIF-OFs, CLIF* consortium organ failure score, *CLIF-C ACLFs* chronic liver failure consortium ACLF.

### Classification and regression tree for 90-day death probability

Rescaled classification scores for each PIRO component was presented in (Table 6). The classification and regression tree was applied to define cut-offs for each score and identify clusters of risk of death, allowing patients’ classification in different risk stages for each variable. Per the cut-offs in the regression tree, each component of the PIRO was categorized into 2 or 3 stages.

**Table 6:** Scoring system of each PIRO component with 90-day mortality.

P	Points	I	Points	R	Points	O	Points
**Age**		**Drug**		**SIRS**		**CLIF-C OFs**	
≤ 50	0	No	0	No	0	6–8	0
> 50	1	Yes	4	Yes	2	9	4
**Gender**		**Variceal bleeding**		**SBP**		10–18	7
Female	0	No	0	No	0		
Male	1	Yes	6	Yes	1		
**ACLF type**		**Infection**		**Pneumonia**			
Type A	0	No	0	No	0		
Type B	6	Yes	2	Yes	8		
Type C	9	**Surgery**					
		No	0				
		Yes	7				

*ACLF* acute-on-chronic liver failure, *SIRS* systemic inflammatory response syndrome, *SBP* spontaneous bacteria peritonitis, *CLIF-OFs, CLIF* consortium organ failure score.

**Figure 3:**
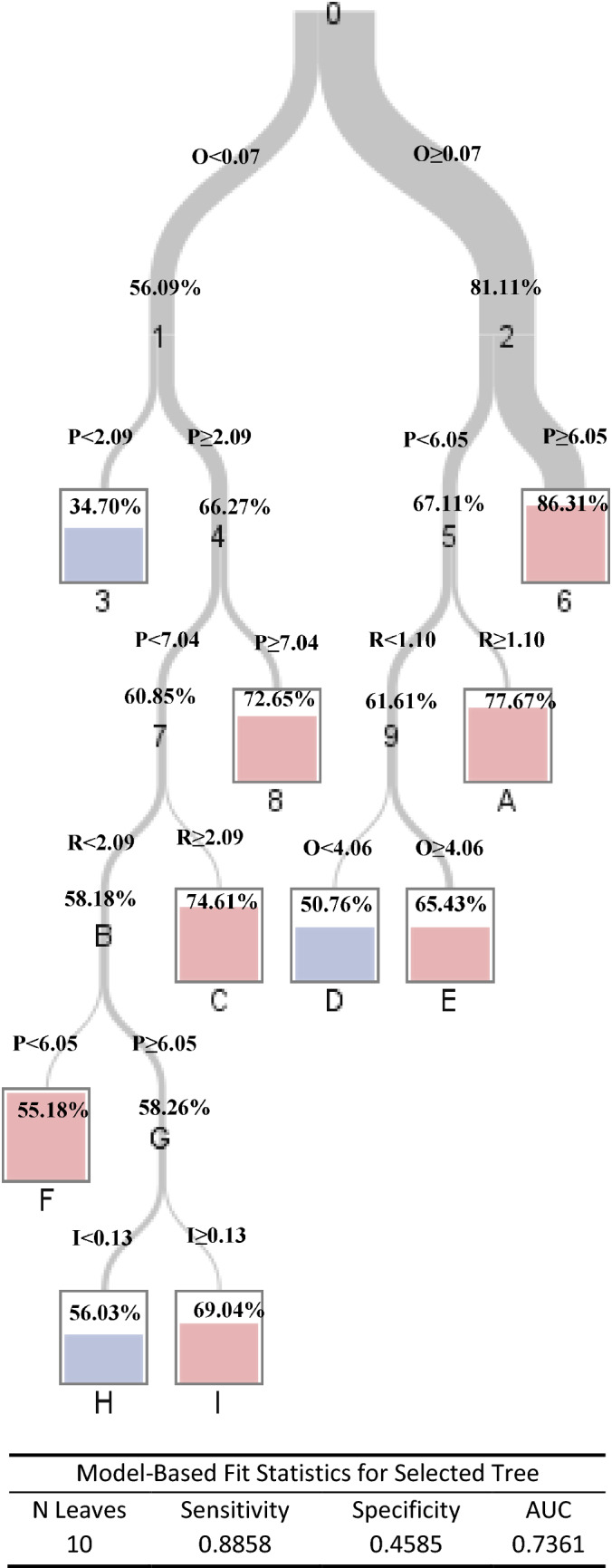
Classification tree for 90-day mortalities. The numbers in the leaves represents for the estimated 90-day mortality. P, Predisposition point calculated from Table 6. I, Injury point calculated from Table 6. R, Response point calculated from Table 6. O, Organ failure point calculated from Table 6.

The classification and regression tree showed different patterns with different cut-offs for each combination of PIRO components. The estimated probabilities of death with each combination of cut-offs of PIRO could obtained from the trees. The sensitivity and specificity of this tree for predicting 90-day death probability were 89% and 46%, respectively (Fig. 3). The positive and negative predictive values of 90-day death probability were 82% and 59%.

### Calculator for each patient calculating the 90-day death probability

Based on the scoring system above, the set of four scores were included in the final combined PIRO model. The sensitivity and specificity of this model for predicting 90-day death probability were 79% and 61% at the biggest Youden index (0.4) with cut-off value of 65%. The positive and negative predictive values of 90-day death probability were 85% and 52%. We used this PIRO model to generate the calculator for each patient to predicting his/her 90-day death probability. Each patient could calculate their estimated 90-day death probability according to the PIRO scoring system using the following formula:

$$90\; \text{day} \; \text{death} \; \text{probability}=\frac{{\text{e}}^{-\left(0.1744\times \text{Ppoint}+0.1746\times \text{Ipoint}+0.1266\times \text{Rpoint}+0.1868\times \text{Opoint}-1.0281\right)}}{1+{\text{e}}^{-\left(0.1744\times \text{Ppoint}+0.1746\times \text{Ipoint}+0.1266\times \text{Rpoint}+0.1868\times \text{Opoint}-1.0281\right)}}$$

## Discussion

The PIRO concept has previously been introduced to describe severity of ACLF^1,19,20^. However, no study had taken a further step to use PIRO concept to establish a prediction model for prediction of ACLF prognosis. This study proposes a prediction model for ACLF patients with HBV infection based on the PIRO concept.

The PIRO concept breakdowns the physiopathology of ACLF, explaining the whole situation of the entity, which could describe more details of the characteristics of patients with ACLF compared to other scoring systems. In our study, we found that the combined PIRO model showed better performance than each PIRO component models, and greater performance than other scoring systems (i.e., MELDs, CLIF-C OFs and CLIF-C ACLFs).

A classification and regression tree presents a convenient way to discriminate patients into different nodes (or clusters) based on their scores of each PIRO component. There were different nodes generated in the trees with different expected mortality rates. According to the PIRO scoring system, each component score of the patient could be calculated, and then the patient could find the corresponding node in the tree according to the scores to estimate his/her probability of death.

Except for the classification tree for predicting 90-day death probability, we also generated a prediction calculator for each patient to calculate his/her own estimated mortality rate according to the four scores in the PIRO scoring system. This calculator was more accurate for predicting each patient’s probability of death, compared to the classification tree which was for a group of patients.

The PIRO prediction model can be easily adopted by clinicians because the parameters estimating the scores are available in standard hospital settings. The model provides computed figures, estimates risk of mortality, and allows easy stratification of patients with clinical and prognostic significance. Meanwhile this study validated the value of the proposal of ACLF type. This model included the novel scoring system of CLIF-C OFs. It clearly improved the predictive ability of the main prognostic scores currently available (i.e., MELDs, CLIF-C OFs, and CLIF-C ACLFs). This new classification tree and calculator have the potential to facilitate HBV related ACLF patient care by making an appropriate management plan based on more accurate risk stratification of patients.

Our study represents the first effort to use the PIRO concept to develop a new prediction model for patients with HBV related ACLF using data from a large prospective cohort. The proposal of ACLF type had not previously been validated. In our study, we validated the higher power of using ACLF type in predicting mortality. Not only did we built a classification tree for stratifying patients with different levels of risk, we also presented a calculator for each patient to calculate his/her own outcome probability.

While the PIRO prediction model generated through the training cohort was validated in the validation cohort, its generalizability to other populations warrants further investigation, particularly for ACLF patients without HBV infection. Nonetheless, it represents a major step towards the clinical application of the PIRO concept, expanding its applicability to all patients with ACLF.

## Supplementary information

Supplementary Figure S1.

Supplementary Table S1.

## Abbreviations

ACLF: Acute-on-chronic liver failure
HBV: Hepatitis B virus
APASL: The Asia–Pacific Association for the Study of the Liver
EASL: European Association for the Study of the Liver
CLIF: Chronic liver failure
AASLD: The American Association for the Study of Liver Diseases
NACSELD: The North American Consortium for the Study of End-Stage Liver Disease
CANONIC study: EASL-CLIF acute on chronic liver failure study
SOFA: Sequential organ failure assessment
CLIF-SOFAs: CLIF-sequential organ failure assessment score
CLIF-C OFs: CLIF consortium organ failure score
CLIF-C ACLFs: CLIF consortium ACLF score
MELD: Model of end-stage liver disease
INR: International normalized ratio
HE: Hepatic encephalopathy
FiO_2_: Fraction of inspired oxygen
MAP: Mean arterial pressure
PaO_2_: Partial pressure of arterial oxygen
SpO_2_: Pulse oximetric saturation
